# Nuclear and cytosolic pS727-STAT3 levels correlate with overall survival of patients affected by clear cell renal cell carcinoma (ccRCC)

**DOI:** 10.1038/s41598-021-86218-x

**Published:** 2021-03-26

**Authors:** Jazmine Arévalo, David Lorente, Enrique Trilla, María Teresa Salcedo, Juan Morote, Anna Meseguer

**Affiliations:** 1 Renal Physiopathology CIBBIM-Nanomedicine, Vall d’Hebron Institut de Recerca (VHIR), Vall d’Hebron 119-129, 08035 Barcelona, Spain; 2 Urology Department, Hospital Universitari Vall d’Hebron (HUVH), Barcelona, Spain; 3 Pathology Department, Hospital Universitari Vall d’Hebron (HUVH), Barcelona, Spain; 4 Biochemistry and Molecular Biology Department, Universitat Autònoma de Barcelona (UAB), Barcelona, Spain

**Keywords:** Tumour biomarkers, Urological cancer

## Abstract

Clear cell renal cell carcinoma (ccRCC) is the most frequent and aggressive subtype of renal carcinoma. So far, the basis of its oncogenesis remains unclear resulting in a deficiency of usable and reliable biomarkers for its clinical management. Previously, we showed that nuclear expression of the signal transducer and activator of transcription 3 (STAT3), phosphorylated at its serine 727 (pS727), was inversely proportional to the overall survival of ccRCC patients. Therefore, in the present study, we validated the value of pS727-STAT3 as a clinically relevant biomarker in ccRCC. This work is a retrospective study on 82 ccRCC patients treated with nephrectomy and followed-up for 10 years. Immunohistochemical expression of pS727-STAT3 was analyzed on a tissue microarray and nuclear and cytosolic levels were correlated with clinical outcome of patients. Our results showed that pS727-STAT3 levels, whether in the nucleus (*p* = 0.002; 95% CI 1.004–1.026) or the cytosol (*p* = 0.040; 95% CI 1.003–1.042), significantly correlate with patients’ survival in an independent-manner of clinicopathological features (Fuhrman grade, risk group, and tumor size). Moreover, we report that patients with high pS727-STAT3 levels who undergone adjuvant therapy exhibited a significant stabilization of the disease (~ 20 months), indicating that pS727-STAT3 can pinpoint a subset of patients susceptible to respond well to treatment. In summary, we demonstrated that high pS727-STAT3 levels (regardless of their cellular location) correlate with low overall survival of ccRCC patients, and we suggested the use of pS727-STAT3 as a prognostic biomarker to select patients for adjuvant treatment to increase their survival.

## Introduction

Clear cell renal cell carcinoma (ccRCC) is the most prevalent and aggressive histological subtype of renal cell carcinoma (RCC) accounting for 80–90% of all malignancies in adult kidney^1,2^. To date, ccRCC is considered the most lethal urological cancer due to its asymptomatic phenotype together with its resistance to chemotherapy and radiotherapy^3,4^. If ccRCC is detected in early stages, partial or radical nephrectomy is the first-line treatment and can prolong survival in 65% of patients. However, 30% of initial organ-confined tumors treated by surgical resection develop local recurrence or metastasis during the follow-up. Conversely, if detection occurs in advanced metastatic stage, there are very limited and no completely effective treatments to date^5–7^. Furthermore, adjuvant therapy has only been approved for metastatic patients, yet less than 50% of cases respond to treatment likely because of the underlying molecular heterogeneity among tumors^8^.

At present, prognosis after surgery has been modestly successful. Classic clinicopathologic features such as Fuhrman grade and tumor-node-metastasis (TNM) stage are the usual predictors of clinical outcome^9,10^, and despite they provide significant information, they are not accurate enough to predict disease progression by themselves. So far, although numerous and extensive studies on the genetic and biochemical features of ccRCC have been performed, there are few usable markers only related to treatment outcome^11^. This scenario poses the urgent need to identify specific biomarkers that can be used for early diagnosis, prognosis and to develop treatment strategies for all ccRCC patients.

In this context, previous studies from our group brought to light the role of the signal transducer and activator of transcription 3 (STAT3) in ccRCC by demonstrating that its activation resulted from the overexpression of the human hepatitis A virus cellular receptor 1 (hHAVCR1), also known as kidney injury molecule 1 (KIM1)^12^. STAT3 is a ubiquitous transcription factor that regulates the expression of hundreds of genes involved in essential biological processes, thus, it is not surprising that its aberrant activation has been related to the onset of more than 50% of all human cancers^13^. Classically, STAT3 activation relies on the phosphorylation of its tyrosine 705 (Y705)^14^, nonetheless, recent reports described the phosphorylation of its serine 727 (S727) as a novel and non-canonical mechanism of STAT3 activation^15,16^. Previously, and in order to establish the role of STAT3 in the ccRCC, our group analyzed the expression of both phosphorylated residues (pY705 and pS727) on localized tumor samples from 98 ccRCC patients who had not undergone chemo- or immunotherapy before or after nephrectomy. Results from that study showed that only nuclear pS727-STAT3 -non pY705- levels were associated with the clinical outcome of ccRCC patients, therefore, it was proposed as an independent factor of overall survival^12^.

In the present study, we evaluated the prognostic value of pS727-STAT3 on a tissue microarray (TMA) from a new cohort of 82 ccRCC patients followed-up for 10 years, and we correlated its expression levels, both in the nucleus and in the cytosol, with the clinical outcome of ccRCC patients. Moreover, the major difference from our previous patients’ cohort^12^ was the inclusion of individuals who underwent tyrosine kinase inhibitors (TKIs) treatment after nephrectomy. These patients allowed the evaluation of the pS727-STAT3 potential to discriminate between those with different responses to adjuvant therapy.

## Results

### Study group

The present study included 56 men (68.3%) and 26 women (31.7%), median age: 72 years range: 38–92, with ccRCC. The tumor was on the right side in 43 cases (52.4%) and on the left side in 39 cases (47.6%). A total of 77 patients (93.9%) presented with localized tumors and 5 (6.1%) with metastasis. Sixty-eight patients (82.9%) underwent radical nephrectomy, while 14 (17.1%) underwent nephron-sparing surgery (tumorectomy or partial nephrectomy). Fuhrman grade grouped I–II consisted of 42 cases (51.2%), and III–IV consisted of 40 cases (48.8%). The most frequently observed tumor size (pT) stages were pT1a in 45.1% and pT1b in 23.2%. Lymphovascular invasion was present in only 3.6% of patients, while the risk group was low in 43.9%, intermediate in 34.1%, and high in 22% of patients studied. Finally, 15 patients (18.3%) who recidivated after surgery, underwent tyrosine kinase inhibitors (TKIs) treatment (Table 1). All patients were considered for statistical analysis. Univariate analysis for cancer-specific survival following kidney surgery was statistically significant for the Fuhrman grade (I–II vs III–IV, *p* = 0.002); risk group (low vs high, *p* = 0.006); and tumor size (1–2 N0 M0 vs 3–4 N1,2 M1, *p* = 0.001) (Table 2).

**Table 1:** Patients and tumor characteristics.

Variable	No. (%) or variable unit
**Patient age (years)**	
Median	72.17
Mean (range)	70.56 (38–92)
**Sex, No. (%)**	
Male	56 (68.3)
Female	26 (31.7)
**Tumor side**	
Right	43 (52.4)
Left	39 (47.6)
**Clinical presentation, No. (%)**	
Radical	68 (82.9)
Neph. sparing	14 (17.1)
Adjuvant treatment, No. (%)	15 (18.3)
**Fuhrman grade, No. (%)**	
I–II	42 (51.2)
III–IV	40 (48.8)
**Primary tumor size, No. (%)**	
pT1a	37 (45.1)
pT1b	19 (23.2)
pT2	4 (4.9)
pT3a	13 (15.9)
pT3b	8 (9.8)
pT4	1 (1.2)
**Lymphovascular invasion, No. (%)**	
pNx	20 (24.4)
pN0	59 (72.0)
pN1	2 (2.4)
pN2	1 (1.2)
**Metastasis, No. (%)**	
pM0	77 (93.9)
pM1	5 (6.1)
**Risk group, No. (%)**	
Low	36 (43.9)
Intermediate	28 (34.1)
High	18 (22.0)
**Follow-up (months)**	
Median for all patients	79.57
Mean for all patients	72.15 (2–112)

*Neph. sparing* nephron-sparing surgery, *pT* tumor size, *pN* nodal involvement, *pM* metastasis.

**Table 2:** Cancer-specific survival following renal surgery.

Variable (grouped)	Univariate analysis mean (95% CI)	*p* value
**Fuhrman grade**		
I–II versus	105.9 (102.5–109.2)	
III–IV	88.3 (77.6–99.0)	0.002
**Risk group**		
Low versus	101.0 (94.5–107.4)	
High	91.7 (80.8–102.5)	0.006
**Primary tumor size (pT)**		
1–2 N0 M0 versus	105.0 (101.2–108.8)	
3–4 N1,2 M1	79.0 (63.6–94.5)	0.001

### ccRCC tumors exhibit different pS727-STAT3 location patterns between the nucleus and the cytosol

**Figure 1:**
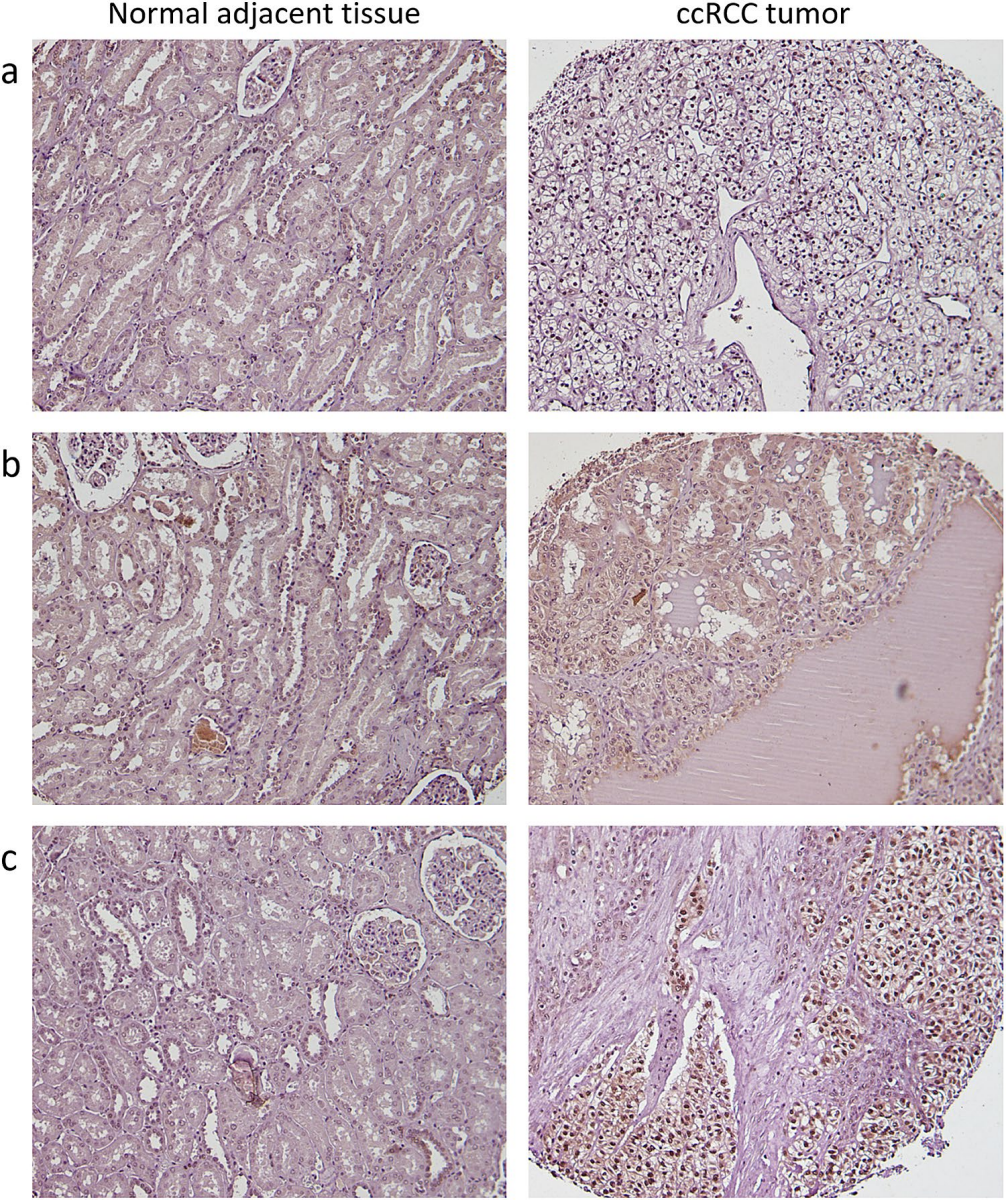
pS727-STAT3 location patterns in ccRCC tumors. Representative images of pS727-STAT3 immunohistochemical analysis showing normal counterparts (left panel) completely negative and tumor samples (right panel) exhibiting positive staining arranged in different location patterns: (**a**) only nuclear pS727-STAT3; (**b**) only cytosolic pS727-STAT3, and (**c**) nuclear and cytosolic pS727-STAT3. Original magnification × 200.

Immunohistochemical analyses showed that unaffected adjacent tissue was negative for pS727-STAT3 staining (Fig. 1, left panel), whereas ccRCC tumors exhibited positive staining both in the nucleus and the cytosol (Fig. 1, right panel). The threshold to distinguish patients with high or low pS727-STAT3 expression was determined as the median value based on the percentage and intensity of stained cells (H-score), thus, cut-off values were set at 80 and 40 (nucleus and cytosol, respectively). According to the H-score, 44 tumors (53.6%) exhibited strong nuclear staining whilst 41 tumors (50%) showed intense cytosolic staining. Conventionally, an activated STAT3, either by Y705 or S727 phosphorylation, is predominantly located within the nucleus regulating gene expression; however, recent studies have demonstrated that STAT3 phosphorylated at its S727 also exerts non-genomic functions in the mitochondria and the endoplasmic reticulum (ER)^16^. These latest findings might explain the presence of pS727-STAT3 in both compartments observed in our study. More interestingly, different expression patterns were also observed among ccRCC tumors where pS727-STAT3 was i) only present in the nucleus (Fig. 1a), ii) completely cytosolic (Fig. 1b), or iii) simultaneously detected in both cellular compartments (Fig. 1c) but without a significant correlation between their independent H-scores (*p* = 0.2). The frequency of these expression patterns is summarized in Table 3. These results suggest that different pS727-STAT3 location patterns could determine different STAT3 functions during ccRCC development and progression.

**Table 3:** Incidence of different pS727-STAT3 expression patterns in ccRCC tumors.

pS727-STAT3	H-score	*n*
Only nuclear	≥ 80	24 (29.2%)
Only cytosolic	≥ 40	21 (25.6%)
Nuclear and cytosolic	≥ 80, ≥ 40	20 (24.3%)

### High nuclear and cytosolic pS727-STAT3 levels identify patients with the lowest overall survival among those with advanced-stage ccRCC disease

**Figure 2:**
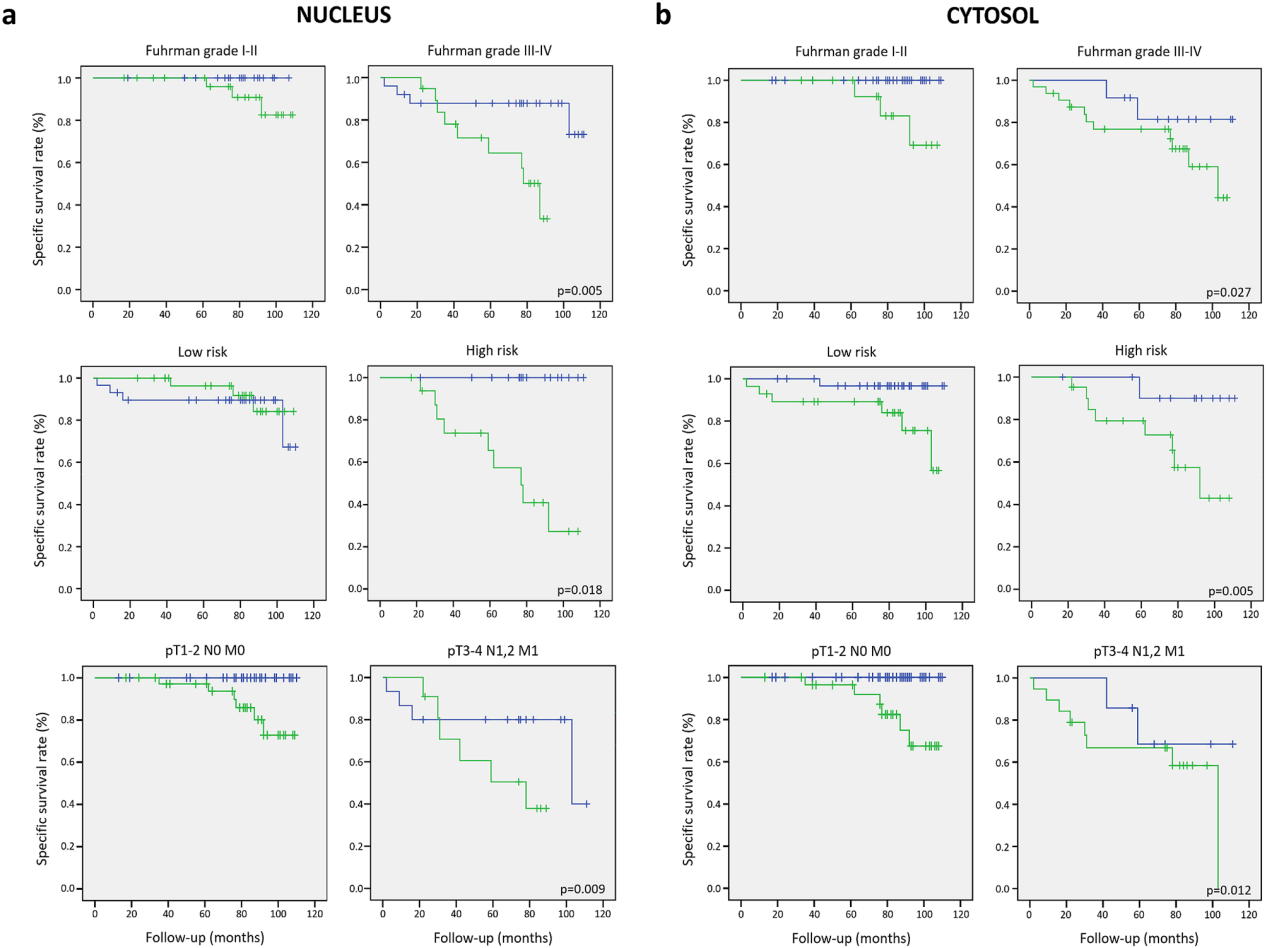
Correlation of pS727-STAT3 H-score with clinical outcome. Kaplan–Meier estimates of 120 months overall survival showing that patients with high pS727-STAT3 H-score (green lines) are prone to die earlier over time when compared to low pS727-STAT3 H-score patients (blue lines), especially those in advanced stages of the disease (Fuhrman grade III–IV, high risk, and tumor size pT3-4 N1,2 M1). Nuclear (blue line, HS < 80; green line, HS ≥ 80) (left panel) and cytosolic (blue line, HS < 40; green line, HS ≥ 40) (right panel) pS727-STAT3 H-score expression levels correlated to mean survival of ccRCC patients according to (**a**) Fuhrman grade (I–II, III–IV), (**b**) risk group (low, high), and (**c**) tumor size (pT1-2 N0 M0, pT3-4 N1,2 M1).

Upon signal intensity evaluation using the H-score, levels of pS727-STAT3 were correlated to the clinical outcome of ccRCC patients. Kaplan–Meier estimates of mortality (120 months overall survival) showed statistically significant differences in overall survival rates between patients in the same advanced-stage clinicopathologic group with high versus low pS727-STAT3 H-score: Fuhrman grade III–IV (nuclear *p* = 0.005, cytosolic *p* = 0.027), high-risk group (nuclear *p* = 0.018, cytosolic *p* = 0.005), and tumor size pT3-4 N1,2 M1 (nuclear *p* = 0.009, cytosolic *p* = 0.012) (Fig. 2a,b). Moreover, multivariate analysis for nuclear (*p* = 0.002; 95% CI 1.004–1.026) and cytosolic (*p* = 0.040; 95% CI 1.003–1.042) pS727-STAT3 H-score indicates that its presence—regardless of its location—significantly correlates with patient survival though cytosolic levels were not statistically as relevant as those in the nucleus (Table 4). Overall, these results demonstrate that high pS727-STAT3 levels—either in the nucleus or in the cytosol—represent a prognostic factor of low overall survival in advanced-stage ccRCC patients independently of the clinicopathological parameters.

**Table 4:** Overall survival of ccRCC patients considering the expression of pS727-STAT3.

H-score	Mean survival (95% CI) (months)	*p* value
**Nuclear pS727-STAT3**		
< 80	107.9 (103.7–112.0)	
≥ 80	88.4 (79.3–97.6)	0.002
**Cytosolic pS727-STAT3**		
< 40	103.0 (95.4–110.7)	
≥ 40	92.9 (85.0–100.9)	0.040

### ccRCC patients with high nuclear expression of pS727-STAT3 exhibit increased disease-free survival after adjuvant treatment

In the present study, patients who received adjuvant treatment (n = 15) in response to recurrence after surgery were found included in the high nuclear pS727-STAT3 levels group (H-score ≤ 80) and advanced stages of the disease according to Fuhrman grade III-IV and tumor size pT3-4, N1,2 M1. Interestingly, the inclusion of these patients in our regression model resulted in a crossover of Kaplan–Meier curves, as observed at the top of the corresponding graphs (Fig. 2a). This particular observation indicates that these patients improved disease-free survival (~ 20 months) after TKIs treatment, and suggests that pS727-STAT3 detection could also be used as a prognostic biomarker for the stratification of patients by indicating which ones (those with high nuclear pS727-STAT3 levels) are susceptible to respond to adjuvant treatment and improve overall survival.

## Discussion

Although multiple risk factors have been associated with the development of ccRCC^17^, to date, the molecular mechanisms behind its etiology are not completely understood which leads to a lack of effective biomarkers clinically usable to predict disease progression or to select patients for particular therapies. So far, the most promising biomarkers for ccRCC are associated with treatment outcome, ranging from clinical parameters and endogenous substances (such as blood pressure or plasma proteins) to pathobiological features specific to individual tumors (such as mutations)^11^. Therefore, the study of molecular markers associated with ccRCC development and progression becomes urgent to provide a better understanding of the molecular pathogenesis of the disease, and consequently, its clinical outcome. In that scenario, a previous study from our group pointed out the role of STAT3 in ccRCC by demonstrating, for the first time, that nuclear presence of its phosphorylated form (at S727) correlated with the overall survival of ccRCC patients^12^. Accordingly, over the past years, it has been extensively confirmed that abnormal activation of STAT3—either by Y705 or S727 phosphorylation—is a key event that contributes to oncogenesis in several tumors other than ccRCC^13^.

Here, we aimed to gain further insight into the value of pS727-STAT3 as a clinically relevant biomarker in ccRCC. Our results demonstrate that pS727-STAT3 levels constitute an independent prognostic factor of overall survival that divides advanced-stage ccRCC patients into two groups: those with low pS727-STAT3 levels and better prognosis, and those with high pS727-STAT3 levels prone to die earlier over time (Fig. 2a,b). Aligned with our results, other studies evaluating STAT3 activation (via pY705 or pS727) on tissue samples of other cancer types such as cervical intraepithelial neoplasia (CIN) and prostate cancer, have also reported a significant correlation of pS727-STAT3 levels to the clinical outcome of those patients^18,19^. These studies along with ours demonstrate that activation of STAT3 through S727 phosphorylation is indeed involved in the development of several malignancies, and suggest that pS727-STAT3 may be responsible for the expression of a subset of genes (different from those regulated by pY705) that promote a more aggressive tumoral phenotype.

Unlike our previous study, the present work included the analysis of cytosolic pS727-STAT3 levels due to recent findings indicating that STAT3 phosphorylation at S727 has an important role in the regulation of mitochondrial activity and modulation of Ca^2+^ release from the ER^16^; both processes in line with the pro-oncogenic role of STAT3. Several studies have demonstrated that mitochondrial STAT3 (mSTAT3), which refers to pS727-STAT3, contributes to the maintenance of energy balance and cell survival under specific cellular stress conditions such as Ras-mediated transformation^20^. Specifically, pS727-STAT3 in the mitochondria has been related to an optimal electron transport chain (ECT) activity, an increase of membrane polarization and ATP production, and enhance of lactate dehydrogenase activity that, consequently, induces aerobic glycolysis and decreases reactive oxygen species (ROS) production^21–23^. In addition to these actions, protection from apoptosis is also triggered by inhibition of the mitochondrial permeability transition pore (MPTP) in collaboration with the ER^24^. In normal conditions, the regulation of Ca^2+^ homeostasis in the ER is mainly under the control of Ca^2+^ pumps and receptors activated by inositol 1,4,5-triphosphate (IP_3_) called IP3R^25^. This process takes place at the mitochondria-associated membranes (MAMs), which locate at the interphase between the mitochondria and the ER^26^. As the membranes of both organelles are nearby, the excessive release of Ca^2+^ mediated by IP3R, in particular IP3R3, can lead to mitochondrial overload, which in turn triggers the opening of the MPTP and the initiation of the intrinsic apoptotic program^27^. Therefore, the ER-mitochondria space acts as a signaling hub for the activity of growth factors, oncogenes, and tumor suppressors that regulate IP3R3 activity^16^. Since both pY705- and pS727-STAT3 localize to the ER and MAMs, phosphoablative mutants (Y705F and S727A) were used to evaluate their effect on IP3R3-mediated Ca^2+^ release founding that only pSer727-STAT3 inhibited Ca^2+^ release by interacting with IP3R3 and facilitating its degradation via the proteasome, thus, avoiding apoptosis^26^.

Our immunohistochemical analysis showed that ccRCC tumors exhibited three different location patterns of pS727-STAT3 between the nucleus and the cytosol. Specifically, pS727-STAT3 was found i) only in the nucleus, ii) only in the cytosol, or iii) in both compartments simultaneously; suggesting that pS727-STAT3 may exhibit different functions at certain stages of tumor development. It might be that pS727-STAT3 controls gene transcription in the nucleus, while regulates energy metabolism in the mitochondria and apoptosis through the ER. Moreover, the presence of pS727-STAT3 in the cytosol, specifically in the mitochondria, might respond to a shift in cellular energy metabolism driving the initial stages of transformation in agreement with the Warburg effect^28^. Otherwise, the presence of pS727-STAT3 in the ER could represent a well-established anti-apoptotic mechanism in the late stages of tumor development. Nevertheless, to confirm this hypothesis, more experiments should be performed to determine whether a correlation between pS727-STAT3 expression pattern and the different stages of ccRCC development actually exists. In line with our data, analysis of pS727-STAT3 levels in CIN samples has also identified nuclear and cytosolic expression of pS727-STAT3, both correlating with nuclear expression of the cellular marker of proliferation Ki67^18^.

Besides the value of pS727-STAT3 as a prognostic factor of overall survival in ccRCC patients, our results also indicate that it may be useful as a novel biomarker to select patients who are candidates for adjuvant therapy. In our cohort, patients who received anti-angiogenic treatment (TKIs) corresponded to those exhibiting metastases or lymphovascular invasion at the time of surgery. In turn, none of the patients with localized tumors received any adjuvant treatment because no information regarding possible benefits for them was available until now. A recent study reported that adjuvant sunitinib versus placebo significantly improved the median duration of disease-free survival in locoregional tumors of ccRCC patients at high risk for tumor recurrence after nephrectomy^29^. Based on this study and our observation that ccRCC patients with a high pS727-STAT3 expression which undergone TKIs treatment experienced a significant stabilization of the disease (~ 20 months), we postulated that determination of pS727-STAT3 levels at the time of surgery could indicate that patients with localized tumors might benefit from adjuvant treatment versus those that only will suffer the adverse effects. We reasoned that the incorporation of molecular markers such as pS727-STAT3 into conventional models (such as Fuhrman grade and TNM stage) might enhance their prognostic accuracy. In that regard, we rationalized that patients in advanced stages of the disease with low pS727-STAT3 expression levels might not need adjuvant treatment because they do not recidivate; however, those patients in the high pS727-STAT3 group could benefit from the treatment. Although the number of patients with adjuvant therapy included in our cohort (n = 15) narrows the interpretation of our results and a larger study is needed, we propose the pS727-STAT3 as a putative biomarker able to indicate treatment after surgery in all advanced-stage ccRCC patients.

In summary, the present study demonstrates that pS727-STAT3 expression, whether in the nucleus or the cytosol, correlates with a low overall specific cancer survival of ccRCC patients and represents an independent prognostic factor to classical clinicopathologic features. Moreover, we propose that measurement of pS727-STAT3 in tumor tissues at the time of surgery can determine which patients shall be treated with currently available adjuvant therapies to increase overall survival. Altogether, pS727-STAT3 represents a biomarker not only for the prognosis of the disease but also for the therapeutic stratification of ccRCC patients.

## Methods

### Case selection

Standard tumor data were obtained from 82 ccRCC patients treated with radical or partial nephrectomy between 2008 and 2010 and clinically followed up to 2017 at the Vall d’Hebron Hospital (Barcelona, Spain). Primary tumor histology was obtained from surgical resections and pathologic information was based on a re-review of all surgical samples to establish histologic subtype.

Clinical and pathologic information was retrospectively reviewed including date of diagnostic, demographics, tumor stage, Fuhrman grade, nodal and metastasis spread, initial and subsequent therapy, as well as outcome information. The anatomic extent of the tumors was classified using the TNM stage system, while prognostic stratification of ccRCC patients was scored using the University of California Los Angeles (UCLA) Integrated Staging System–*Union Internationale Contre le Cancer* (UICC) nomogram. Clinical and follow-up information was recorded by physician reports. Because of the availability of novel targeted therapies since 2008, patients who recidivated were treated with adjuvant therapy, nonetheless, all of them were included in the present study.

### Tissue microarray (TMA)

The TMA was constructed from paraffin-embedded samples of 82 ccRCC patients provided by the Department of Pathology at the Vall d’Hebron Hospital (Barcelona, Spain). One section from each specimen was stained with hematoxylin and eosin and reviewed for the selection of representative areas. For each sample, 3 representative areas of 1 mm diameter cores were obtained using a semi-automated tissue arrayer (Chemicon International). As controls, 3 cores of adjacent benign tissue far from the tumor were included. Finally, tissue cores were paraffin-embedded in a spaced array pattern^30^.

Informed consent from all participants was obtained for the use of all human tissues in this study following the rules of the Spanish Biomedical Research Law. The ethics committee of the Hospital Universitari Vall d’Hebron (HUVH) approved the study protocol and the methodology was performed following the relevant guidelines and regulations.

### TMA immunohistochemistry

Four-micrometer-thick sections from TMA blocks were cut using a microtome, deparaffinized overnight (ON) at 55 °C, and hydrated into milli-Q H_2_O through sequential steps of graded ethanol. The immunohistochemistry (IHC) was performed using the EnVision + Dual Link System-HRP (DAB+) kit (Dako #K4065) according to the supplier's instructions. Antigen retrieval was performed with sodium citrate buffer pH 6.0 (Dako #S2031) for 15 min. Endogenous peroxidase activity was quenched followed by unspecific binding blocking with 5% normal horse serum (Sigma Aldrich # H0146) for 1 h at room temperature (RT). For pS727-STAT3 staining, TMAs were incubated ON at 4 °C with primary antibody α-pS727-STAT3 (Cell Signaling #9134) at a final dilution of 1:50. The secondary antibody (labeled polymer-HRP mouse/rabbit) was incubated for 1 h at RT. Finally, HRP detection with DAB-Chromogen and hematoxylin (Sigma Aldrich #HHS32) counterstaining were performed at RT for 20 min and 1 min, respectively. As a negative control, the primary antibody was replaced with non-immune bovine serum (Alpha Diagnostics # 20001-2-1).

### TMA evaluation

pS727-STAT3 staining was scored by two renal pathology experts (MTS and ITR), blinded to clinicopathologic variables and fully independent to each other. pS727-STAT3 expression was evaluated in a semi-quantitative manner by immune-histo-score (H-score) based on the percentage and intensity of stained cells as follows: 0 = no appreciable staining; 1 = weak staining; 2 = moderate staining; and 3 = strong staining. Therefore, the H-score was calculated as 1 × (% weak) + 2 × (% moderate) + 3 × (% intense) ranging from 0 to 300 ^31^. The inter-rater reliability between experts was substantial by having a Cohen’s kappa of 0.75 for nuclear and 0.73 for cytosolic H-scores. For each ccRCC sample, 3 independent tissue cores were evaluated presenting an average coefficient of variance (CV) of 23.4%, therefore, H-score was expressed as the mean.

### Statistical analyses

Correlations between pS727-STAT3 expression and clinicopathologic parameters were evaluated with the nonparametric Mann–Whitney U test. Kaplan–Meier survival curves were compared using the log-rank test and multivariate analysis was carried out using a Cox regression model to estimate the independent prognostic importance of clinicopathologic parameters. Concordance between experts’ scoring was determined using Cohen’s kappa statistic^32^. Correlation between nuclear and cytosolic H-scores was defined using linear regression, while the correlation between replicates was assessed by calculating the coefficient of variance among samples. All the statistical analysis was performed with the Statistical Package for Social Sciences software (SPSS, IBM).

## Data Availability

The datasets analyzed during the current study are not publicly available due to privacy policies. Data are however available from the corresponding author on reasonable request and with permission of the Spanish Biomedical Research Law and the ethics committee of the Hospital Universitari Vall d’Hebron (HUVH).
